# The Discovery and Discoverers of Anæsthesia

**Published:** 1877-05

**Authors:** 


From the May number of the *Virginia Medical Monthly*, we quote the following summary of facts, set forth in a historic sketch of the discovery and discoverers of anæsthesia, by J. Marion Sims, M.D., New York:

"Now let us summarize the facts set forth in the foregoing historic sketch. We know,

"1st. That since 1800 the inhalation of nitrous oxide gas produced a peculiar intoxication, and even allayed headache and other minor pains.

"2d. That Sir Humphrey Davy proposed it as an anæsthetic in surgical operations.

"3d. That for more than fifty years the inhalation of sulphuric ether has been practiced by the students in our New England Colleges as an excitant, and that its exhilarating properties are similar to those of nitrous oxide gas.

"4th. That the inhalation of sulphuric ether, as an excitant, was common in some parts of Georgia forty-five years ago, though not practised in the colleges.

"5th. That Wilhite was the first man to produce profound anæsthesia, which was done accidentally with sulphuric ether, in 1839.

"6th. That Long was the first man to intentionally produce anæsthesia for surgical operations, and that was done with sulphuric ether, in 1842.

"7th. That Long did not by accident hit upon it, but that he reasoned it out in a philosophical and logical manner.

"8th. That Wells, without any knowledge of Long's labors, demonstrated, in the same philosophic way, the great principle of anæsthesia by the use of nitrous oxide gas (1844).

"9th. That Morton intended to follow Wells in using the gas as an anæsthetic in dentistry, and for this purpose asked Wells to show him how to make the gas (1846).

"10th. That Wells referred Morton to Jackson for this purpose, as Jackson was known to be a scientific man and an able chemist.

"11th. That Morton called on Jackson for information on the subject, and that Jackson told Morton to use sulphuric ether instead of nitrous oxide gas, as it was known to possess the same properties, was as safe, and easier to get.

"12th. That Morton, acting upon Jackson's off-hand suggestion, used the ether successfully in the extraction of teeth (1846).

"13th. That Warren and Hayward and Bigelow performed important surgical operations in the Massachusetts General Hospital—October, 1846—on patients etherized by Morton, and that this introduced and popularized the practice throughout the world.

"In Boston, Mass., a monument has been erected to the discoverer of anæsthesia, but no man is designated thereon by name. The citizens of Hartford, Conn., have erected a bronze statue of Wells—by Bartlett—in their Capitol Park, claiming for him the discovery of anæsthesia. This is as it should be. We have no objection to it; and would suggest that the names of Long, Wells, Morton, and Jackson be inscribed on the Boston column—one on each side—as co-discoverers of anæsthesia. The State of Georgia will, at no distant day, erect at its Capitol or its University, a statue of Long, who was unquestionably the first discoverer of anæsthesia.

"All the claimants of the honor of discovering anæsthsia are Americans. To each is due a certain measure of credit, but no one man can claim this great honor exclusively. The names of Long, Wells, Morton, and Jackson will doubtless be associated as co-laborers in the great work, and to these must be added the immortal name of Sir James Y. Simpson, who introduced chloroform and enlarged the domain of anæsthesia.

"Sir James received the highest honor from his government in recognition of the great service he had rendered humanity. I wish we could say the same of our benefactors and government. Our great republic leaves our discoverers and scientists to rest in obscurity and to starve.

"Long lost his all during our great civil war, and in his old age he is now being worked to death for the daily bread necessary to support himself and family.

"The fate of Wells, Morton, and Jackson is most pitiable.

"Wells, disappointed in carrying off the honor of the great discovery of anæsthesia, became insane and committed suicide in New York, in 1848.

"Morton, disappointed at not receiving a pecuniary recognition from Congress for his labors, fretted himself into a congestion of the brain. In July, 1868, he returned to New York from Washington in the wildest state of excitement. Fatigue, anxiety, and sleepless nights had exhausted his vital powers. Dr. Lewis A. Sayre and Dr. Yale were called to him on the 15th July. They considered his condition as critical, placed him in the hands of a trained nurse, ordered leeches to his temples, cups on the spine, and ice to his head. Dr. Morton would not submit to treatment. As soon as Dr. Sayre left, he ordered his buggy to go to the Riverside Hotel, saying he knew he would soon be well if he could get out of the hot city. He drove furiously up Broadway, and through the Central Park. At the upper end of the Park he leaped from his buggy and ran to a lake near by to cool his burning brain. Being persuaded to get into his buggy again, he drove a short distance, then leaped out, and jumping over a fence, he fell down in a state of insensibility. He was then taken moribund to St. Luke's Hospital, where he died an hour or two later.

"Jackson has been for some time in an insane asylum, hopelessly incurable.

"How mournful the fate of these remarkable men! How sad to think that their lives were embittered with envy, jealousy and uncharitableness towards each other! Let us forget their faults, and remember only the good that has resulted from their labors.

"It is said that "The evil that men do lives after them." But here the good that these men did will live after them, and the evil forever.

"Vaccinnation is perhaps the greatest boon ever conferred by science on humanity. Anæsthesia is the next. England gave us the one. America the other. England recognized the labors of Jenner, not, however, in a manner commensurate with the magnitude of his work. America should recognize the labors of Long, Wells, Morton, and Jackson, if not in a manner commensurate with the value of their work, at least to such an extent as to relieve the necessities of their several families, thereby proving that republics are not always ungrateful. Government aid, voluntarily tendered at this time, would be acceptable to all of them, for they are all really in need of it. Each of these families ought to receive at least one hundred thousand dollars.

"I propose, then, that the whole medical profession, North, South, East and West, unite in asking Congress, at its next session, to appropriate this sum, as an anæsthesia fund to be divided equally between the families of Long, Wells, Morton, and Jackson.

"One hundred thousand dollars is a small sum to offer where men have sacrificed their lives for the good of the whole civilized world, leaving their families in straightened circumstances. How small this pittance when measured by the benefits these men conferred on the world!

"Let us, as Americans, rise above all party, all prejudice, all sectionalism, and demand of the Government this appropriation for the great work accomplished by these martyrs to science and humanity."

